# UK national clinical audit: management of pregnancies in women with HIV

**DOI:** 10.1186/s12879-017-2255-6

**Published:** 2017-02-20

**Authors:** S. Raffe, H. Curtis, P. Tookey, H. Peters, A. Freedman, Y. Gilleece, on behalf of the British HIV Association Audit and Standards Sub-Committee, Ann Sullivan, Brian Angus, David Asboe, Garry Brough, Fiona Burns, David Chadwick, Duncan Churchill, Valerie Delpech, Katja Doerholt, P. Gupta, Aoife Molloy, Julie Musonda, Chinyere Okoli, Ed Ong, Alison Rodger, Michael Rayment, Caroline Sabin

**Affiliations:** 1 British HIV Association, London, UK; 2 National Study of HIV in Pregnancy and Childhood, University College London, London, UK

**Keywords:** HIV, Mother to child transmission, Vertical transmission

## Abstract

**Background:**

The potential for HIV transmission between a pregnant woman and her unborn child was first recognized in 1982. Since then a complex package of measures to reduce risk has been developed. This project aims to review UK management of HIV in pregnancy as part of the British HIV Association (BHIVA) audit programme.

**Methods:**

The National Study of HIV in Pregnancy and Childhood (NSHPC), a population-based surveillance study, provided data for pregnancies with an expected delivery date from 1/1/13 - 30/6/14. Services also completed a survey on local management policies. Data were audited against the 2012 BHIVA pregnancy guidelines.

**Results:**

During the audit period 1483 pregnancies were reported and 112 services completed the survey. Use of dedicated multidisciplinary teams was reported by 99% although 26% included neither a specialist midwife nor nurse. 17% of services reported delays >1 week for HIV specialist review of women diagnosed antenatally. Problematic urgent HIV testing had been experienced by 9% of services although in a further 49% the need for urgent testing had not arisen. Delays of >2 h in obtaining urgent results were common.

Antiretroviral therapy (ART) was started during pregnancy in 37% women with >94% regimens in accordance with guidelines. Late ART initiation was common, particularly in those with a low CD4 count or high viral load.

Eleven percent of services reported local policy contrary to guidelines regarding delivery mode for women with a VL <50 copies/mL at ≥36 weeks. According to NSHPC reports 27% of women virologically eligible for vaginal delivery planned to deliver by CS.

**Conclusions:**

Pregnant women in the UK are managed largely in accordance with BHIVA guidelines. Improvements are needed to ensure timely referral and ART initiation to ensure the best possible outcomes.

**Electronic supplementary material:**

The online version of this article (doi:10.1186/s12879-017-2255-6) contains supplementary material, which is available to authorized users.

## Background

The potential for HIV transmission from a pregnant woman to her unborn child was recognized in 1982, prior even to the identification of the HIV virus [1]. Since this time significant advances have been made in our knowledge of methods to reduce this risk. The dramatic reduction in transmission rate in the UK and Ireland from 25.6% in the 1990s [2] to 0.5% in 2011 [3] demonstrates the success of these interventions.

Prevention of mother to child transmission (MTCT) is complex: interventions aim to reduce risk throughout pregnancy, labour, delivery and the neonatal period. Consequently a specialist multi-disciplinary team, including HIV clinicians, midwives, obstetricians and paediatricians is a valuable tool. Approximately 1300 women with HIV have given birth annually in the UK since 2004 [4]. Areas of concentrated prevalence exist, particularly within London, while for some centres caring for a pregnant woman with HIV is a rare event.

In addition to preventing HIV transmission, implementing these interventions is also beneficial for maternal health. Universal antenatal screening facilitates diagnosis of those living with unknown HIV. Pregnancy can also act as a trigger to review management, improve engagement and provide additional care to those with a known HIV diagnosis. Regular specialist multi-disciplinary review can help identify any pregnancy or HIV related complications and also provide psychological support where required.

Surveillance of paediatric HIV and HIV in pregnancy has been ongoing in the UK and Ireland since 1986 and 1989 respectively, through the mechanisms of the National Study of HIV in Pregnancy and Childhood (NSHPC), described in detail elsewhere [3].

Since 2001 the British HIV Association (BHIVA) has produced guidelines to support clinicians in the delivery of care aimed at reducing MTCT. These guidelines have been reviewed regularly and updated. The aim of this audit was to review the UK management of HIV in pregnancy in comparison with the 2012 BHIVA guidelines.

## Methods

NSHPC provided BHIVA with data for pregnancies with an expected date of delivery between 1/1/13 and 30/6/14. The data included demographic and clinical details, timing of HIV diagnosis, viral load, CD4 count and antiretroviral treatment (ART) in pregnancy, whether sexual health screening had been offered in pregnancy, and mode of delivery; no patient identifiers were provided to BHIVA. In addition, pregnancy-lead clinicians for all HIV services throughout the UK were invited by the BHIVA audit group to complete a survey detailing local arrangements for the management of pregnancy in HIV. They were encouraged to complete the survey with input from local HIV-lead obstetricians and paediatricians (Additional file 1).

Pseudonymised data were stored and analysed in a password protected, encrypted Microsoft excel spreadsheet. Data were audited against the 2012 BHIVA guidelines for the management of HIV in pregnancy [5]. Ethical approval and informed consent were not required as this study was a clinical audit based on routinely collected data [6].

## Results

1483 pregnancies were reported during the audit period and 112 HIV services completed the survey (Table 1). The 1483 pregnancies occurred in 1469 women, the majority of whom were of Black African ethnicity (1089, 73.7%). 1251 women (85.2%) acquired HIV through heterosexual sex, 17 (1.2%) via injecting drug use and 21 (1.4%) as a consequence of MTCT. Route of transmission was unknown in 180 (12.3%). Median age at expected date of delivery was 33 years (range 16 – 50).

Of the 1483 pregnancies, 1263 (85.2%) occurred in women already known to be HIV positive at conception, 217 (14.6%) were to women diagnosed with HIV during their pregnancy and in three (0.2%) cases timing of HIV diagnosis was unclear (Table 2). Of the 1263 pregnancies in women with a pre-existing HIV diagnosis, 920 (73%) conceived while established on antiretroviral therapy (ART). An HIV viral load was available between 0 and 16 weeks gestation in 612 women (66.5%) who conceived on ART of whom 87.7% (537) were suppressed to ≤50 copies/mL. Of the 217 women diagnosed in the index pregnancy, 198 (91.2%) were diagnosed in the antenatal clinic, 14 were diagnosed elsewhere and in five cases place of diagnosis was not reported. The majority of antenatal diagnoses (140, 65%) were made before 16 weeks gestation. Only seven (3%) women were diagnosed after 36 weeks gestation: six following late pregnancy booking including one woman who presented in labour. In one case a woman declined antenatal testing but was later diagnosed by non-maternity services following presentation with symptomatic HIV.

**Table 1:** Summary of survey results

Does your service have a dedicated HIV in pregnancy MDT?		
	Yes	111 (99%)
	No	1 (1%)
Does this MDT include:		
	An HIV physician	112 (100%)
	An obstetrician	111 (99%)
	A paediatrician	110 (98%)
	An HIV midwife	29 (26%)
	An HIV clinical nurse specialist	21 (19%)
Following a new HIV diagnosis during antenatal screening, how quickly would you expect a women to be seen in the HIV clinic?		
	Same/next day	40 (36%)
	2 – 3 days	23 (21%)
	Within a week	29 (26%)
	1 - 2 weeks	19 (17%)
	Not answered	1 (1%)
Do you have a policy for the use of ART in pregnancy?		
	Yes	109 (97%)
	No	2 (2%)
	No response	1 (1%)
Would you use raltegravir in a women presenting after 28 weeks gestation with a VL > 100,000 copies/mL?		
	Use routinely	56 (50%)
	May use	43 (38%)
	No policy/has not arisen	11 (10%)
	Would not use	1 (1%)
	Not sure	1 (1%)
What arrangement do you have in place for urgent HIV testing for women presenting in labour, with ruptured membranes of requiring delivery with no result?		
	Arrangement for urgent lab test	95 (85%)
	Point of care testing in all delivery units	15 (13%)
	Urgent lab test not available	1 (1%)
	Not sure	1 (1%)
Have you experienced problems with urgent HIV testing?		
	Problems experienced	10 (9%)
	Provided without problems	40 (36%)
	Need not arisen	55 (49%)
How long does it take to obtain an urgent HIV laboratory test result?		
	>2 h in working hours	21 (19%)
	>2 h outside of working hours	56 (50%)
Do you have a policy on mode of delivery in HIV?		
	Yes	107 (96%)
	No	2 (2%)
	Not sure/not answered	3 (3%)
What mode of delivery would you recommend for those on ART with a VL <50 copies/mL at >36 weeks, and no relevant obstetric factors?		
	Planned vaginal delivery	95 (85%)
	Maternal choice	9 (8%)
	Pre-labour caesarean section	3 (3%)
	Other/not answered	5 (5%)

*MDT* multidisciplinary team, *ART* antiretroviral therapy

### Multidisciplinary teams

BHIVA guidelines recommend the use of dedicated multidisciplinary teams in the delivery of antenatal HIV care. As a minimum these teams should comprise an HIV specialist, obstetrician, specialist midwife and paediatrician. One hundred and eleven (99%) services reported a dedicated multidisciplinary team (MDT) although 29 (26%) included neither a specialist midwife nor nurse. Nineteen (17%) centres reported delay of > 1 week between women being diagnosed with HIV via routine antenatal screening and receiving specialist HIV assessment.

### Urgent HIV testing

BHIVA recommends urgent HIV testing in cases where a woman without a documented HIV test result presents in labour, following rupture of membranes or with other reasons to expedite delivery. Ninety-five (84.8%) HIV services reported arrangements in place for urgent laboratory testing although only 15 (13.4%) services reported access to point of care testing (POCT). Problems with urgent HIV testing were reported by 10 (8.9%) services although in a further 55 (49.1%) services the need for urgent testing was not reported to have arisen. Delays of >2 h in obtaining urgent test results were reported by 21 (18.8%) services in working hours and 56 (50%) out of hours.

### Antiretroviral therapy

One hundred and nine (97.4%) services reported the use of a local ART in pregnancy policy. Two services did not have a local policy and one service did not respond. In 920 (62.5%) pregnancies the woman was taking ART when she conceived. In this situation BHIVA recommends that women should remain on their current regimen unless on protease inhibitor monotherapy or a regimen containing stavudine (d4T) or didanosine (DDI). Data regarding continuation of established ART regimen was not available although this figure is likely to be high.

In 552 pregnancies ART was started after conception. The BHIVA-recommended ART regimens vary depending on maternal need for ART (a CD4 cell count ≤350 cells/mm^3^ was used as a surrogate marker in this audit as full clinical information was not available) or if the main indication for treatment is to prevent MTCT. Baseline maternal viral load (VL) also impacts on choice of agent. Overall accordance with BHIVA recommended regimens was very high at 93% in those requiring ART for maternal health and over 98% for those requiring ART solely to prevent MTCT (Table 3). Information regarding previous ART treatment was not available so in some cases deviation from guidelines could be explained by previous failed ART regimens.

**Table 2:** Baseline characteristics

	Number of women/pregnancies^a^ (%)
Ethnicity	
Black African	1083 (73.7%) women
White	250 (17%) women
Black Caribbean	46 (3.1%) women
Other/ not stated	90 (6.1%) women
Age at EDD^b^	
16 – 19	12 (0.8%) women
20 – 29	344 (23.4%) women
30 – 39	952 (64.8%) women
40 +	161 (11%) women
HIV acquisition	
Heterosexual	1251 (85.2%) women
MTCT	21 (1.4%) women
Injecting drug use	17 (1.2%) women
Other/ not stated	180 (12.3%) women
Timing of HIV diagnosis	
Pre-conception	1263 (85.2%) pregnancies
During pregnancy	217 (14.6%) pregnancies
Not stated	3 (0.2%) pregnancies
ART status	
Conceived on ART	920 (62%) pregnancies
Conceived off ART	549 (37%) pregnancies
Unclear	14 (0.9%) pregnancies

^a^1483 pregnancies in 1469 women were reported in the audit period

^b^First EDD for women with two pregnancies

BHIVA guidelines recommend that women requiring ART for maternal health should commence treatment as soon as possible and within two weeks of diagnosis. Of the 214 women requiring ART for maternal health, 105 (%) were diagnosed during pregnancy. Of these women only 30 (29%) commenced ART within the two-week target with 25 (23.8%) starting 15 – 28 days following diagnosis and 43 (41.%) starting >28 days. In seven cases (6.6%) the start date was not recorded. Delay in treatment initiation cannot be explained solely by concerns regarding ART use in the first trimester as only 25/68 (36.8%) women were diagnosed in this period.

BHIVA recommend that all women starting ART during pregnancy should start treatment by the beginning of week 24 gestation and those with a VL >30,000 copies/mL by 16 weeks gestation. Data regarding timing of initiation was available in 523 pregnancies: 402 (76.6%) with a baseline VL <30,000 copies/mL (those with an unreported baseline VL were also included in this group) and 121 (23.4%) with a baseline VL >30,000 copies/ml. Of the 402 pregnancies where ART should have been initiated by week 24, 318 (79.1%) were managed in accordance with guidelines and 84 (20.9%) started ART after 24 weeks. Compliance with guidance was worse in the 121 pregnancies where ART should have commenced by week 16, where only 47 (38.8%) started ART on time, 56 (46.3%) started between 16 – 24 weeks gestation and 18 (14.9%) did not start until after 24 weeks. Of the 158 pregnancies where ART initiation was late, only 54 could be attributed to late booking, two to seroconversion and two to both seroconversion and late booking (Table 4).

**Table 3:** Choice of antiretroviral regimen

CD4 count (cells/mm^3^)	Viral load (copies/mL)	BHIVA recommendation^a^	Number of women	In accordance with recommendation
≤350	-	TVD/FTC, ABC/3TC or ZDV/3TC + EFV, NVP or bPI	214	93%
>350	>100,000	TFV/FTC, ABC/3TC or ZDV/3TC + bPI	11	100%
	10,000 – 100,000	As above or ZDV/3TC/ABC	81	99%
	<10,000	As above or ZDV monotherapy	162	98%
	unknown	-	84	-

*TVD* truvada, *FTC* emtricitabine, *ABC* abacavir, *3TC* lamivudine, *ZDV* zidovudine, *EFV* efavirenz, *NVP* nevirapine, *bPI* boosted protease inhibitor

^a^Raltegravir was accepted as an alternative to EFV/NVP/bPI when started after the first trimester

**Table 4:** Timing of antiretroviral initiation

Subgroup	BHIVA recommendation	Number of women	Number (%) in accordance with recommendation
CD4 ≤ 350^a^	Within 14 days of HIV diagnosis	105	30 (28.6)
VL <30,000^b^ copies/mL	By week 24 gestation	402	318 (79.1)
VL >30,000 copies/mL	By week 16 gestation	121	47 (38.8)

^a^Women diagnosed with HIV during antenatal period

^b^Also includes women who conceived off ART where viral load was not reported

### Mode of delivery

At point of analysis 1354 pregnancies had resulted in a live birth, five in stillbirth and in 124 pregnancies the outcome had yet to be reported. Planning for appropriate mode of delivery is an important factor in the prevention of MTCT and recommendations are dependent on the maternal VL at or after 36 weeks. Despite this a VL at this time point was reported in only 613 (45%) pregnancies. Only 106 women delivered prior to 36 weeks. The 2012 BHIVA guidelines recommend vaginal delivery for women on ART with an HIV VL <50 copies/mL at 36 weeks gestation. When surveyed, 95/112 (84.8%) HIV services stated they would plan for a vaginal delivery in this circumstance, nine (8%) stated they would be guided by maternal choice, three (2.7%) would plan for an elective caesarean and five (4.5%) did not respond. In the audit period 540 women had a VL of <50 copies at ≥36 weeks of whom 391 (72%) planned a vaginal delivery, 148 (27%) a caesarean and one (<1%) had no reported plan. Where women have a VL of 50–399 copies/mL at ≥36 weeks, BHIVA recommend that a vaginal delivery be considered taking individual factors into account. Fifty women fell into this category of whom 24 (48%) planned a vaginal delivery and 26 (52%) a caesarean section. All women with a VL ≥400 copies/mL at ≥36 weeks are advised to have a caesarean section. Twenty-four women were in this group, 19 (79.2%) of whom planned for a caesarean section, three (12.5%) initially planned for vaginal delivery but had caesarean sections, and two (8.3%) did not have a plan as one woman was diagnosed with HIV in labour and the second woman did not attend for antenatal care prior to the onset of labour.

Actual mode of delivery was vaginal in 630 (46.5%), via caesarean section in 719 (53.1%) and not reported in 5 (0.4%) live births. Emergency caesarean section rates were high, occurring in 249 (28.0%) of those planning a vaginal delivery, 88 (20.9%) of those planning an elective caesarean section and in 26 (60.5%) of women with no reported plan. Three women delivered vaginally with a VL ≥400 copies/mL at >37 weeks, comprising the two late presenters mentioned above and a woman who planned caesarean section on ART but had a spontaneous vaginal delivery. A further 26 women delivered vaginally for whom VL data were not available.

## Discussion

### Main findings

This audit has provided a valuable review of the current management of HIV in pregnancy in the UK. The results have been largely encouraging, particularly regarding the use of multi-disciplinary teams and choice of antiretroviral regimen.

While the vast majority of women were treated with an antiretroviral regimen in accordance with guidelines, the frequent delay seen in initiating treatment is of concern. Only 29% of women diagnosed in pregnancy with a maternal need for ART were started within the recommended two weeks. This leaves a significant proportion of women at increased and unnecessary risk of HIV related morbidity and mortality. In addition this audit has identified important failings in starting ART by recommended gestational cut-offs, particularly where the viral load was very high. It is well recognized that maternal viral load at delivery is the best predictor of perinatal transmission [7, 8]. The need for timely initiation of ART was demonstrated by a multicenter UK study where only 46% of the women with a VL in the upper quartile (>32,641 copies/mL) achieved viral suppression when commencing ART at a median gestation of 23 weeks [9]. Any perceived risk of congenital abnormality as a consequence of early ART has not been confirmed by research studies. The NSHPC reviewed rates of congenital abnormality occurring in 8576 pregnancies between 1990 and 2007. It found an abnormality rate of 2.8%, consistent with that seen nationally [10].

The reported poor access to reliable urgent HIV testing is also of major concern. While uptake of antenatal screening, introduced as a universal recommendation in 2000, is consistently high at >90% in all regions throughout the UK [11], it is essential that units are able to confirm HIV status in cases where a woman presents late or where an earlier test has been declined. Intrapartum ART is a valuable tool in late diagnosis [12, 13] and knowledge of a mother’s status also ensures access to neonatal post-exposure prophylaxis and follow-up. The use of POCT was particularly low at 13.4%. A further survey exploring the barriers to use of POCT would be valuable as expanding availability of POCT may help to solve some of the logistical issues units face in providing rapid testing out of hours.

### Strengths and limitation

This strength of this audit lies in the very high rates of reporting to the NSHPC, allowing insight into practice throughout the UK including units based in low HIV prevalent areas. Nevertheless, there have been limitations to this audit. Lack of reported viral load data was particularly significant in reviewing planned mode of delivery where this measurement was only reported in 49% (613/1248) of pregnancies that had reached 36 weeks gestation at time of analysis. We were also unable to access either medical or obstetric history which may have given insight into decisions regarding antiretroviral therapy or mode of delivery that did not follow BHIVA recommendations.

Data for this audit was obtained from the standard data collected by the NSHPC rather than via additional means. This was advantageous as response rates to the NSHPC are very high and therefore reflect national practice well and it also removed the need for additional and time-consuming data collection by the individual services. However, as a consequence of this some information that would have allowed for greater interpretation of the results is lacking. For example the NSHPC do not routinely collect the previous ART history from women and therefore it was not possible to establish if some women were started on ART that deviated from the guidelines as a consequence of previous failed regimens or drugs. Likewise it is not possible to explain why delays in initiating ARVs were seen and whether this can be attributed to referral pathways, a lack of urgency or the need for additional pre-treatment diagnostics.

Interpreting the high reported rates of both planned and elective caesarean is difficult due to the lack of obstetric history. The proportion of second or subsequent pregnancies in women with HIV is increasing and many women will have had a previous caesarean section in accordance with earlier guidelines. Early evidence that an elective caesarean section reduced risk of HIV transmission by >50% [14] has since evolved with the introduction of combination ART and multiple studies have since shown the rate of MTCT to be <0.5% irrespective of delivery mode when VL <50 copies/mL [15, 16]. BHIVA guidelines have evolved accordingly and since 2005 have recommended that vaginal delivery should be considered in all those with a VL <50 copies/mL at or after 36 weeks gestation. In 2012 this was expanded to allow consideration of vaginal delivery for those with a VL <400 copies/mL considering actual VL, trajectory of VL, ART duration and adherence and the woman’s views [5].

## Conclusion

This audit demonstrates a need for services to review their ability to access urgent HIV testing, to strengthen referral pathways between general and specialist antenatal care services and to identify where delays in starting women on ART are occurring. It is also important to ensure that local policy is kept up to date with national guidance to ensure women are able to access the best possible care available.

## Additional file

Additional file 1: List of BHIVA Audit participants. The supplementary document lists the units across the United Kingdom that completed the survey detailing local management of HIV in pregnancy. (DOCX 130 kb)

## Abbreviations

ART: Antiretroviral therapy
BHIVA: British HIV Association
D4T: Stavudine
DDI: Didanosine
MDT: Multidisciplinary team
MTCT: Mother to child transmission
NSHPC: National Study of HIV in Pregnancy and Childhood
POCT: Point of care test
VL: Viral load
