# Supplementary material for: UK national clinical audit: management of pregnancies in women with HIV
Source: BMC Infect Dis. 2017 Feb 20;17:158. doi: 10.1186/s12879-017-2255-6 (PMC5319092; doi:10.1186/s12879-017-2255-6)
Supplement: Additional file 1: — List of BHIVA Audit participants. The supplementary document lists the units across the United Kingdom that completed the survey detailing local management of HIV in pregnancy. (DOCX 130 kb) [file 12879_2017_2255_MOESM1_ESM.docx]

**BHIVA Audit participants**

The BHIVA Audit and Standards Sub-Committee would like to thank clinicians at the following services which took part in the survey, as well as the National Study of HIV in Pregnancy and Childhood and all those who submitted patient data:

Area Infection Unit, Monklands Hospital, Airdrie
Department of GU Medicine, Ysbyty Gwynedd Hospital, Bangor
Department of Integrated Sexual Health and HIV Medicine, Barking Community Hospital, Barking
Clare Simpson Clinic, Barnet General Hospital, Barnet
Department of GU Medicine, Barnsley Hospital NHS Foundation Trust, Barnsley Department of GU Medicine, Royal United Hospital, Bath
Department of GU Medicine, Beckenham Hospital, Beckenham
Department of GU Medicine, Bedford Hospital, Bedford
Department of GU Medicine, Royal Hospitals Trust, Belfast
Department of HIV Medicine, Queen Elizabeth Hospital, Birmingham
Department of GU Medicine, Blackburn Royal Infirmary, Blackburn
Blackpool Sexual Health Services, Whitegate Drive Health Centre, Blackpool
The Pannel Suite Clinic, Royal Bournemouth Hospital, Bournemouth
Department of GU Medicine, Bradford Hospitals NHS Trust, Bradford
Lawson Unit (HIV) Outpatients, Royal Sussex County Hospital, Brighton
Department of GU Medicine, Queen's Hospital, Burton-upon-Trent
Bury Sexual Health Service, Townside Primary Care Centre, Bury
Department of GU Medicine, West Suffolk Hospital, Bury St Edmunds
Department of GU Medicine, Frimley Park Hospital, Camberley
Infectious Diseases & HIV/GU Medicine Clinic 1A, Addenbrooke's Hospital NHS Trust, Cambridge
The Gate Clinic, Kent & Canterbury Hospital, Canterbury
Infectious Diseases Unit, University Hospital of Wales, Cardiff
Department of GU Medicine, Cardiff Royal Infirmary, Cardiff
Department of GU Medicine, St Helier Hospital, Carshalton
The Blanche Heriot Unit, St Peter's Hospital, Chertsey
Fletcher Unit of Sexual Health, St Richard's Hospital, Chichester
Department of GU Medicine, Essex County Hospital, Colchester
Sexual & Reproductive Health Clinic, Crawley Hospital, Crawley
Department of GU Medicine, Croydon University Hospital, Croydon
Department of GU Medicine, Derbyshire Royal Infirmary NHS Trust, Derby
Department of GU Medicine, Dewsbury and District Hospital, Dewsbury
Department of Sexual Health, Avenue House Sexual Health Clinic, Eastbourne Department of GU Medicine, Lothian University Hospitals, Edinburgh
Exeter NHS Walk-in Centre, Exeter

Department of GU Medicine, Falkirk & District Royal Infirmary, Falkirk
Department of GU Medicine, Gartnaval General Hospital, Glasgow
Department of GU Medicine, Gloucestershire Royal Hospital, Gloucester
The Riverside Clinic, Gravesham Community Hospital, Gravesend
Department of GU Medicine, Bure Clinic, James Paget Hospital NHS Trust, Great Yarmouth Department of GU Medicine, Stirling Street Medical Centre, Grimsby

Department of GU Medicine, Northwick Park Hospital, Harrow
Sexual Health Clinic, Station Plaza Health Centre, Hastings
Department of GU Medicine, Wycombe General Hospital, High Wycombe Department of GU Medicine, Huddersfield Royal Infirmary, Huddersfield The Oak Tree Centre, Huntingdon

Highland Sexual Health, Raigmore Hospital, Inverness
Orwell Clinic, Ipswich
Department of Sexual Health, West Middlesex University Hospital, Isleworth
Ashwood Centre for Sexual Health, St Mary's Hospital, Kettering
Department of Infectious Diseases, Worcestershire Acute Hospitals NHS Trust, Kidderminster
Department of GU Medicine, Queen Elizabeth Hospital, King's Lynn
The Wolverton Centre, Kingston Hospital, Kingston upon Thames
Department of Sexual Health, Whytemans Brae Hospital, Kirkcaldy
Department of GU Medicine, Leeds General Infirmary, Leeds
Department of Haematology, Leeds Teaching Hospitals NHS Trust, Leeds
Tropical and Infectious Disease Unit, Royal Liverpool University Hospital, Liverpool Department of GU Medicine, Royal Glamorgan Hospital, Llantrisant
Caldecot Centre, King's College Hospital, London
Department of GU Medicine/HIV, Newham University Hospital NHS Trust, London Department of GU Medicine/HIV, North Middlesex Hospital, London
Trafalgar Clinic, Queen Elizabeth Hospital, London
Centre for Sexual Health and HIV Research, Mortimer Market Centre, London
Department of HIV Medicine, Royal Free London NHS Foundation Trust, London Department of GU Medicine, Courtyard Clinic, St George's Hospital, London
Jefferiss Wing Centre for Sexual Health, Imperial College Healthcare NHS Trust, London Patrick Clements Clinic, Central Middlesex Hospital, London
HIV/GUM Directorate: St Stephen's Centre, Chelsea and Westminster Hospital, London
The Ambrose King Centre, The Royal London Hospital, London
Department of GU Medicine, University Hospital Lewisham, London
Department of GU Medicine, Luton & Dunstable Hospital NHS Trust, Luton
Department of GU Medicine, Macclesfield District Hospital, Macclesfield
Rubin Clinic, Department of GU Medicine, Maidstone Hospital, Maidstone
Department of Infectious Diseases, North Manchester General Hospital, Manchester Department of GU Medicine, Withington Hospital, Manchester
Department of GU Medicine, Manchester Royal Infirmary, Manchester
Department of Sexual Health and HIV, North Manchester General Hospital, Manchester Department of Infectious Diseases, James Cook University Hospital, Middlesbrough Department of Infection & Tropical Medicine, Royal Victoria Infirmary, Newcastle upon Tyne

Department of GU Medicine, Cordell Centre, Royal Gwent Hospital, Newport
Department of Sexual Health, Northampton General Hospital, Northampton
Department of GU Medicine, Grove Clinic, Norfolk & Norwich University Hospital, Norwich Department of Infectious Diseases, Nottingham City Hospital, Nottingham
Oldham Sexual Health Service, Royal Oldham Hospital, Oldham
Department of GU Medicine, Oxford Radcliffe NHS Trust, Oxford
Sexual Health Service, Pembroke Dock Healthcare Centre, Pembroke Dock
Department of Sexual Health, Peterborough and Stamford NHS Foundation Trust, Peterborough
Department of GU Medicine, Derriford Hospital, Plymouth
Department of Sexual Health, Glan Clwyd District General Hospital NHS Trust, Rhyl
Nye Bevan House, Pennine Acute Trust, Rochdale
Department of GU Medicine, Rotherham NHS Foundation Trust, Rotherham
Department of GU Medicine, Hospital of St Cross, Rugby
Sexual Health Department, Salisbury District Hospital, Salisbury
Department of GU Medicine, Royal Hallamshire Hospital, Sheffield
Department of Infection, Inflammation and Immunity, Royal Hallamshire Hospital, Sheffield HIV/GUM Directorate, Upton Hospital, Slough
Department of GU Medicine, Ealing Hospital, Southall
Department of GU Medicine, Southport & Ormskirk NHS Trust, Southport
Department of Microbiology and GU Medicine, Jersey General Hospital, St Helier
Kingsway Health Centre, Stevenage
Sexual Health Services, Staffordshire and Stoke on Trent Partnership, Stoke-on-Trent Department of GU Medicine, Sunderland Royal Hospital, Sunderland
Department of GU Medicine, Sherwood Forest Hospitals NHS Trust, Sutton-in-Ashfield Department of GU Medicine, Singleton Hospital, Swansea
Department of Sexual Health, Great Western Hospitals NHS Foundation Trust, Swindon Department of GU Medicine, Musgrove Park Hospital, Taunton
Department of GU Medicine, Royal Cornwall Hospital, Truro
Department of GU Medicine, The Hillingdon Hospital, Uxbridge
Josephine Butler Centre, King Street Health Centre, Wakefield
Walsall Integrated Sexual Health Service, Manor Hospital, Walsall
Watford Sexual Health Centre, West Hertfordshire Hospital NHS Trust, Watford Department of GU Medicine, Southend Hospital, Westcliffe on Sea
Department of GU Medicine, Weymouth Community Hospital, Weymouth
Department of GU Medicine, Arrowe Park Hospital, Wirral
Department of GU Medicine, New Cross Hospital, Wolverhampton
Department of GU Medicine, Wrexham Maelor Hospital, Wrexham
